# Epoch Analysis of On-Treatment Disability Progression Events over Time in the Tysabri Observational Program (TOP)

**DOI:** 10.1371/journal.pone.0144834

**Published:** 2016-01-15

**Authors:** Heinz Wiendl, Helmut Butzkueven, Ludwig Kappos, Maria Trojano, Fabio Pellegrini, Dominic Paes, Annie Zhang, Shibeshih Belachew, Tysabri Observational Program (TOP) Investigators

**Affiliations:** 1 Department of Neurology–Inflammatory Disorders of the Nervous System and Neurooncology, University of Münster, Münster, Germany; 2 Department of Medicine, Royal Melbourne Hospital, University of Melbourne, Victoria, Australia; 3 Melbourne Brain Centre, Royal Melbourne Hospital, University of Melbourne, Victoria, Australia; 4 Department of Neurology, Box Hill Hospital, Monash University, Victoria, Australia; 5 Neurology, Department of Medicine, University Hospital Basel, Basel, Switzerland; 6 Neurology, Department of Clinical Research, University Hospital Basel, Basel, Switzerland; 7 Neurology, Department of Biomedicine, University Hospital Basel, Basel, Switzerland; 8 Department of Neuroscience and Sense Organs, University of Bari, Bari, Italy; 9 Biogen, Cambridge, Massachusetts, United States of America; University Hospital of Heidelberg, GERMANY

## Abstract

**Objective:**

To evaluate the effect of natalizumab on disability progression beyond 2 years of treatment in clinical practice.

**Methods:**

Analyses included the 496 relapsing-remitting multiple sclerosis (RRMS) patients among 5122 patients in the Tysabri Observational Program (TOP) who had completed 4 continuous years of natalizumab treatment and had baseline (study enrollment) and postbaseline Expanded Disability Status Scale (EDSS) assessments. Proportions of patients with 6-month or 12-month confirmed ≥1.0-point EDSS progression relative to baseline were compared in treatment months 1–24 and 25–48. Sensitivity analyses compared progression rates in months 13–24 and 25–36.

**Results:**

Baseline characteristics appeared similar between the overall TOP population (N = 5122), patients who had completed 4 years of natalizumab treatment (n = 469), and patients eligible to complete 4 years in TOP who had discontinued natalizumab after 2 years of treatment (n = 514). Among 4-year completers, the proportion of patients with 6-month and 12-month confirmed EDSS progression decreased between months 1–24 and 25–48 of natalizumab treatment by 42% (from 10.9% to 6.3%; *p* < 0.01) and 52% (from 9.5% to 4.6%; *p* < 0.01), respectively. Few patients had 6-month or 12-month confirmed EDSS progression in both epochs (0.6% and 0.2%, respectively). Between months 13–24 and 25–36 of treatment, the proportion of patients with 6-month and 12-month confirmed EDSS progression decreased by 60% (from 7.5% to 3.0%; *p* < 0.01) and 58% (from 6.7% to 2.8%; *p* < 0.01), respectively. Significant reductions in disability progression events between months 13–24 and 25–36 were also observed in relapse-free patients.

**Conclusion:**

In this observational study, the disability progression rate decreased further beyond 2 years of natalizumab treatment. Patients who responded well and remained on continuous natalizumab therapy for over 4 years had sustained and potentially enhanced reductions in EDSS progression over time.

## Introduction

In the phase 3 natalizumab safety and efficacy in relapsing-remitting multiple sclerosis (RRMS) clinical trial (AFFIRM), natalizumab reduced the risk of 3-month and 6-month confirmed disability progression over 2 years by 42% and 54%, respectively, compared with placebo [1]. Moreover, a larger proportion of natalizumab-treated patients had no evidence of clinical or radiologic disease activity in year 2 (68%) than in year 1 (47%) of the AFFIRM trial [2]. Comparisons of natalizumab efficacy across treatment epochs before and beyond 2 years have not been reported.

The 10-year, prospective Tysabri (natalizumab) Observational Program (TOP; NCT00493298) was established to report the long-term safety and efficacy of natalizumab for the treatment of RRMS in a clinical practice setting. In the recent 5-year interim analysis of TOP data, mean Kurtzke Expanded Disability Status Scale (EDSS) scores of natalizumab-treated patients were unchanged over 5 years [3].

The availability of long-term disability data from TOP allows for an investigation of the effects of natalizumab on disability progression rates in various treatment epochs. In a post hoc multiple-event analysis of TOP EDSS data, we tested the hypothesis that the effect of natalizumab on disability progression could change over time, in particular beyond 2 years, by comparing the rate of confirmed EDSS progression events in the same patient population across treatment epochs ranging up to 4 years.

## Methods

### Patients

A detailed description of TOP study methods has previously been published [3]. To qualify for the study, patients had to meet local eligibility criteria for natalizumab treatment and have received <4 natalizumab infusions prior to enrollment. In order to reduce the possible bias in the comparison between treatment epochs by selective depletion of patients at risk, only patients who completed at least 4 years of natalizumab treatment in TOP were assessed in this post hoc analysis (database lock May 1, 2013) [4–6]. Patients were also required to have both baseline and postbaseline EDSS assessments.

### Study design

TOP is an ongoing, open-label, multinational, observational study of patients initiating natalizumab treatment for RRMS. Patients receive 300 mg natalizumab intravenously every 4 weeks. Clinical assessments are performed every 6 months. The TOP study protocol was approved by each center’s independent ethics committee. The study design is in accordance with the Declaration of Helsinki and Good Clinical Practice guidelines, and all enrolled patients provided written informed consent.

#### EDSS progression multiple-event analyses

In this post hoc multiple-event analysis, EDSS progression events were compared in the same patient population across time epochs. Baseline EDSS score was reported by the physician at enrollment. Any EDSS score that was ≥1.0 point over baseline with confirmation ≥6 or ≥12 months later was counted as an EDSS progression event. For patients who had a progression event in the first epoch, a new baseline was established, defined as the last EDSS score available from the first epoch, and assessment of EDSS progression events in the second epoch was performed in reference to that new baseline rather than the original baseline at enrollment. If any of a patient’s EDSS scores met the criteria for an EDSS progression event within an epoch, the patient was counted as having progressed in that epoch. Confirmation of progression events was allowed to occur after the epoch had ended with no limitation in time, except in sensitivity analyses as described below.

Epoch analyses of EDSS progression event occurrence were restricted to patients who completed 4 years of natalizumab treatment in TOP (the 4-year completer population) to prevent biases due to selective discontinuation of nonresponders to treatment, which is inherently present in observational studies and could have induced a misinterpreted apparent increase of efficacy over time [4–6]. To determine if disability progression event comparisons within the 4-year completer population were likely to be representative of other patient populations in TOP, baseline characteristics and disability assessments were compared with the overall TOP population and the patient population who were expected to complete 4 years based on first dose date but instead discontinued natalizumab treatment after 2 years. Patients who discontinued treatment within the first 2 years were not considered in this analysis, since their time on therapy was insufficient to provide reliable data about disability progression events occurring even within the first treatment epoch.

#### Treatment epochs

Initial comparisons of natalizumab treatment effects on disability progression events within the 4-year completer population were performed using 2-year epochs (months 1–24 vs. months 25–48). It was recognized that this analysis had 2 potential biases: (1) because the 2-year treatment epochs were based on the first natalizumab dose and TOP permitted patients to enroll after as many as 3 doses, 3 months of treatment and observation time could have been lost in the first 2-year epoch, which could bias results against observing an increase in treatment efficacy in the subsequent 2 years, and (2) because not all of the 4-year completers had ≥12 months of additional observation time beyond 4 years to allow for confirmation of EDSS progression events occurring in year 4, the opportunity for confirmation of EDSS progression differed between the epochs for some patients, which could bias results toward observing an increase in treatment efficacy in the second epoch. To prevent these potential biases from significantly affecting the overall analysis, an additional analysis was performed to compare only EDSS progression events in months 13–24 with those in months 25–36.

#### Sensitivity and subgroup analyses

A sensitivity analysis was performed to keep the time period for progression confirmation consistent across the month 13–24 and month 25–36 epochs. The analysis censored the confirmation of progression events occurring after month 36 (day 1008) and month 48 (day 1344) for the month 13–24 and month 25–36 epochs, respectively.

Sensitivity analyses for the comparison of EDSS progression events between months 13–24 and 25–36 were also conducted to assess the contribution of potential confounding factors. To test the contribution of baseline EDSS score, a new baseline EDSS assessment was used (the first EDSS assessment occurring between months 6 and 12). To determine if the findings were impacted by relapse-associated EDSS progression or EDSS improvement, analyses were performed after excluding patients who relapsed or who had confirmed EDSS improvement. As in the TOP study protocol, relapses were defined as new or recurrent neurologic symptoms, not associated with fever, lasting ≥24 hours, and followed by a 30-day period of stability or improvement.

A subgroup analysis comparing proportions of patients with confirmed 6-month and 12-month disability progression by baseline EDSS score (<3.0 and ≥3.0) was performed to assess the relationship between baseline disability status and confirmed progression over time.

### Statistical analyses

Descriptive statistics were used to summarize baseline characteristics and EDSS score changes over time for each of the specified populations. Patient proportions with disability progression in each epoch and between TOP patient populations were compared using McNemar tests. Annualized relapse rate in each epoch was estimated using a negative binomial model.

## Results

### Study population

Of 5122 patients enrolled in TOP (database lock May 1, 2013), 501 had ≥4 years of natalizumab treatment. Of those, 5 were missing either baseline or postbaseline EDSS assessments. Thus, 496 patients were included in the 4-year completer population for this post hoc analysis. Out of a total of 1282 patients who could have completed 4 years of natalizumab treatment in TOP based on first-dose date, 267 discontinued treatment in the first 2 years and 514 discontinued treatment after 2 years (S1 Fig). Sixty-two of the 267 patients who discontinued in the first 2 years (23%) and 85 of the 514 patients who discontinued after 2 years (17%) reportedly did so due to lack of efficacy.

**Fig 1:**
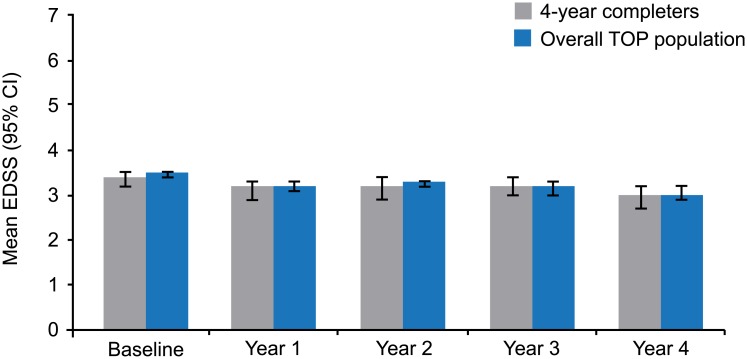
Yearly mean EDSS scores in 4-year completers (n = 496) and the overall TOP population (N = 5122). For the overall TOP population, all patients with EDSS measured within the indicated year were included regardless of whether or not they completed treatment over that year. EDSS: Expanded Disability Status Scale; TOP: Tysabri Observational Program.

**Fig 2:**
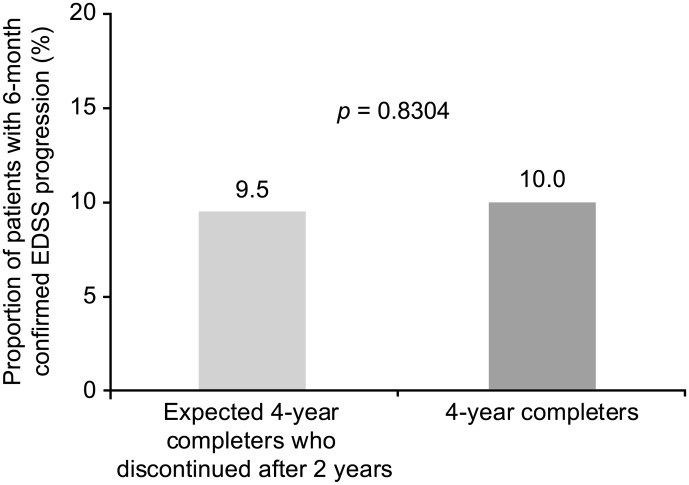
Proportion of patients with 6-month confirmed EDSS progression during the first 2 years among the 4-year completers (n = 496) and expected 4-year completers who discontinued after 2 years (n = 514). EDSS: Expanded Disability Status Scale.

No differences were observed in baseline characteristics between the 4-year completer population, the overall study population, and the population of patients who were expected to complete 4 years on May 1, 2013, based on enrollment date but instead discontinued natalizumab treatment after 2 years (Table 1). Mean EDSS scores during each of 4 years of TOP were very similar between the 4-year completer population and the total TOP population (Fig 1). The proportion of patients with 6-month confirmed EDSS progression at 2 years did not differ significantly between the 4-year completers (10.0%) and the patients who were expected to complete 4 years based on first-dose date but discontinued natalizumab treatment after 2 years (9.5%) (*p* = 0.8304) (Fig 2). However, the cumulative probability of 6-month confirmed EDSS progression in the first 2 years of TOP appeared higher in those patients who discontinued due to reported lack of efficacy (Kaplan-Meier [KM] estimate = 22%) than in either the 4-year completer population (KM estimate = 11%) or patients who discontinued for reasons other than lack of efficacy (KM estimate = 10%).

**Table 1:** Baseline characteristics.

Baseline Characteristic	Overall Population (N = 5122)	4-Year Completers (n = 496)	Expected 4-Year Completers Who Discontinued After 2 Years (n = 514)
Age, years, mean (SD)	37.1 (9.7)	37.0 (9.4)	38.1 (9.7)
Gender, % female	72	76	67
No. of relapses in prior year, mean (SD)	2.0 (1.0)	2.0 (1.1)	2.0 (1.1)
No. of relapses in prior year, n (%)			
≤1 relapse	1807 (35)^a^	178 (36)	176 (34)
>1 relapse	3310 (65)^a^	318 (64)	338 (66)
EDSS score, mean (SD)	3.5 (1.6)	3.4 (1.6)	3.5 (1.7)
EDSS score, n (%)			
<3.0	1900 (37)^b^	184 (37)	181 (35)
≥3.0	3190 (63)^b^	312 (63)	330 (65)
Disease duration, years, median (range)	7.3 (0–43.9)^c^	7.5 (0.2–42.7)	9.0 (0.2–38.2)
No. of prior DMTs, n (%)			
0	473 (9)	46 (9)	36 (7)
1	2330 (45)	261 (53)	258 (50)
≥2	2319 (45)	189 (38)	220 (43)
Treatment^d^ duration prior to natalizumab, years			
Mean (SD)	4.1 (3.7)	3.7 (3.4)	4.1 (3.5)
Median (range)	3.0 (0–26.5)	2.9 (0–21.1)	3.1 (0–20.1)
No. of natalizumab doses prior to TOP enrollment, n (%)			
0	2182 (43)	202 (41)	181 (35)
1	1153 (23)	91 (18)	109 (21)
2	926 (18)	72 (15)	112 (22)
3	857 (17)	131 (26)	112 (22)

DMT, disease-modifying treatment; EDSS, Expanded Disability Status Scale; SD, standard deviation; TOP, Tysabri Observational Program.

^a^n = 5117;

^b^n = 5090;

^c^n = 5097;

^d^MS treatment other than natalizumab.

### EDSS progression rate over time by 2-year treatment epochs

**Fig 3:**
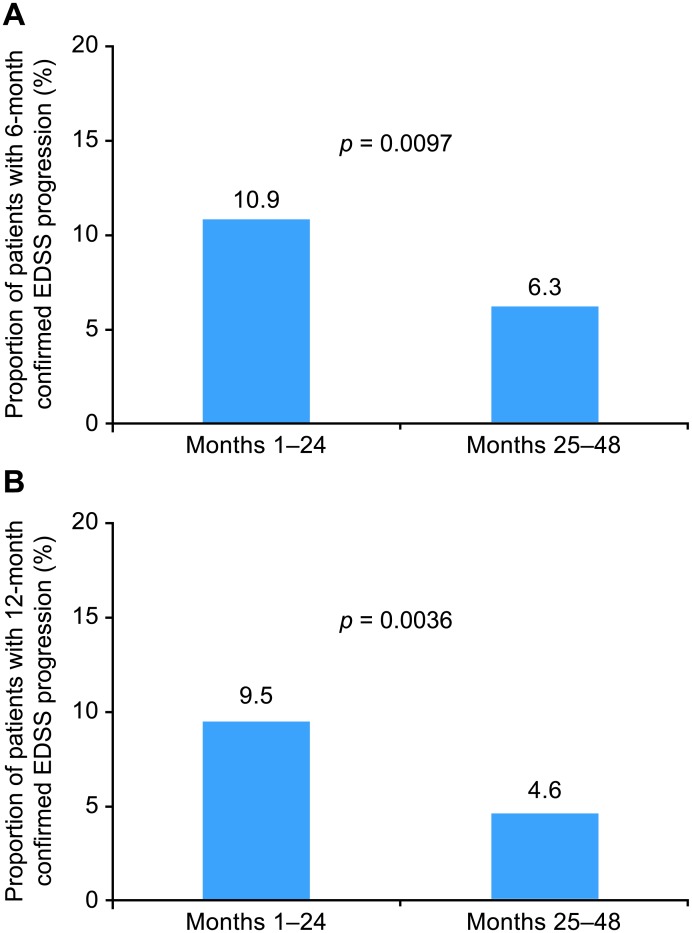
Proportion of patients with (A) 6-month and (B) 12-month confirmed EDSS progression during months 1–24 compared with months 25–48.

The proportions of patients with 6-month and 12-month confirmed EDSS progression were compared between months 1–24 and months 25–48 of natalizumab treatment. Of the 496 4-year completers, a significantly higher proportion of patients had 6-month confirmed EDSS progression in months 1–24 (54 of 496 [10.9%]) than in months 25–48 (31 of 496 [6.3%]; *p* = 0.0097), corresponding to a 42% relative reduction in 6-month confirmed disability progression after the first 2 years of natalizumab treatment (Fig 3A). Likewise, a significantly higher proportion of patients had 12-month confirmed EDSS progression in months 1–24 (47 of 496 [9.5%]) than in months 25–48 (23 of 496 [4.6%]; *p* = 0.0036), corresponding to a 52% relative reduction in 12-month confirmed disability progression after the first 2 years of natalizumab treatment (Fig 3B).

**Fig 4:**
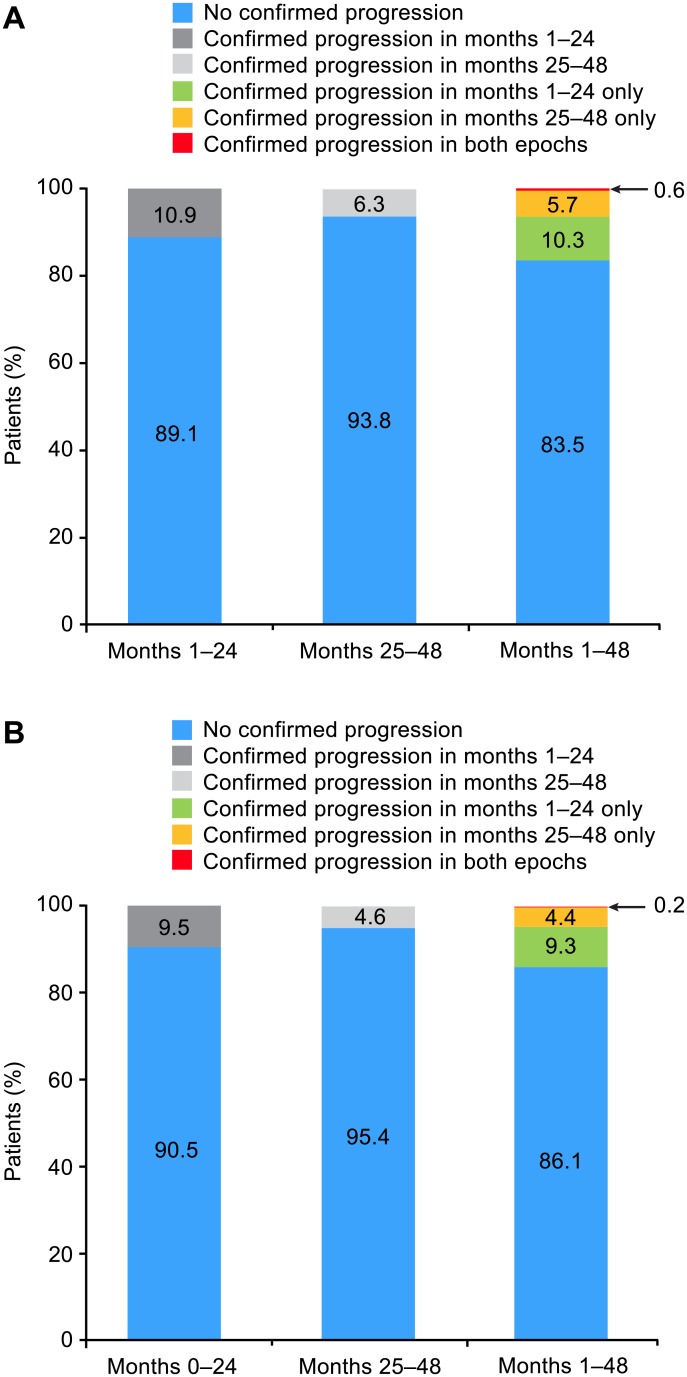
Proportion of patients with (A) 6-month and (B) 12-month confirmed EDSS progression in different treatment epochs.

The great majority of patients who experienced confirmed disability progression in the first 2 years remained free of disability progression in the next 2 years (Fig 4). Of the 4-year completers with 6-month confirmed EDSS progression between months 1 and 24 (n = 54), 94% (n = 51) were free of subsequent progression between months 25 and 48. Of the 4-year completers with 12-month confirmed EDSS progression between months 1 and 24 (n = 47), 98% (n = 46) were free of subsequent progression between months 25 and 48. Only 3 of 496 patients (0.6%) experienced 6-month confirmed disability progression in both treatment epochs, and only 1 of 496 patients (0.2%) experienced 12-month confirmed disability progression in both treatment epochs.

### EDSS progression between months 13–24 and 25–36

**Fig 5:**
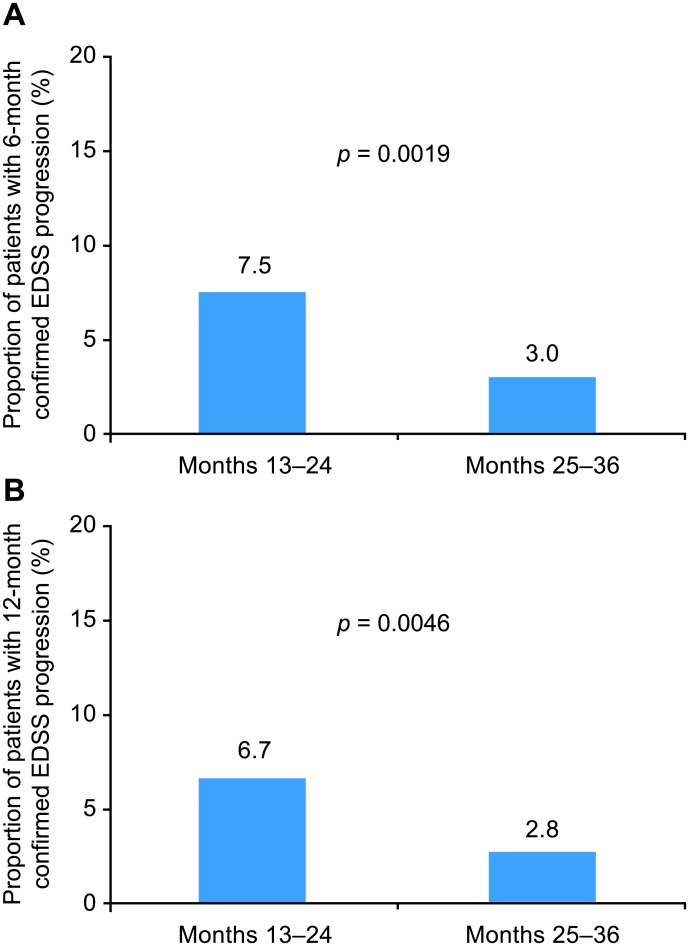
Proportion of patients with (A) 6-month and (B) 12-month confirmed EDSS progression during months 13–24 compared with months 25–36.

An analysis of EDSS progression in the month 13–24 and month 25–36 epochs was performed, as these were the most reliable windows for assessing yearly changes in disability with natalizumab treatment on a yearly basis. A significantly higher proportion of patients had 6-month confirmed EDSS progression in months 13–24 (37 of 496 [7.5%]) than in months 25–36 (15 of 496 [3.0%]; *p* = 0.0019; Fig 5A). The proportion of patients with 12-month confirmed EDSS progression was also significantly higher in months 13–24 (33 of 496 [6.7%]) than in months 25–36 (14 of 496 [2.8%]; *p* = 0.0046; Fig 5B). Thus, between months 13–24 and 25–36 of natalizumab treatment, the rate of 6-month and 12-month confirmed EDSS progression was reduced by 60% and 58%, respectively.

The proportion of patients who experienced 6- or 12-month confirmed EDSS progression in both treatment epochs (months 13–24 and months 25–36) was 0.2% (1 of 496 patients).

A sensitivity analysis that restricted the window for confirmation of progression to 12 months following the end of the treatment epoch showed similar results. Six-month confirmed EDSS progression occurred in 37 of 496 patients (7.5%) in months 13–24 and in 13 of 496 patients (2.6%) in months 25–36 (*p* = 0.0005), corresponding to a 65% relative reduction. Twelve-month confirmed progression occurred in 31 of 496 patients (6.3%) in months 13–24 and in 10 of 496 patients (2.0%) in months 25–36 (*p* = 0.0008), corresponding to a 68% relative reduction.

**Fig 6:**
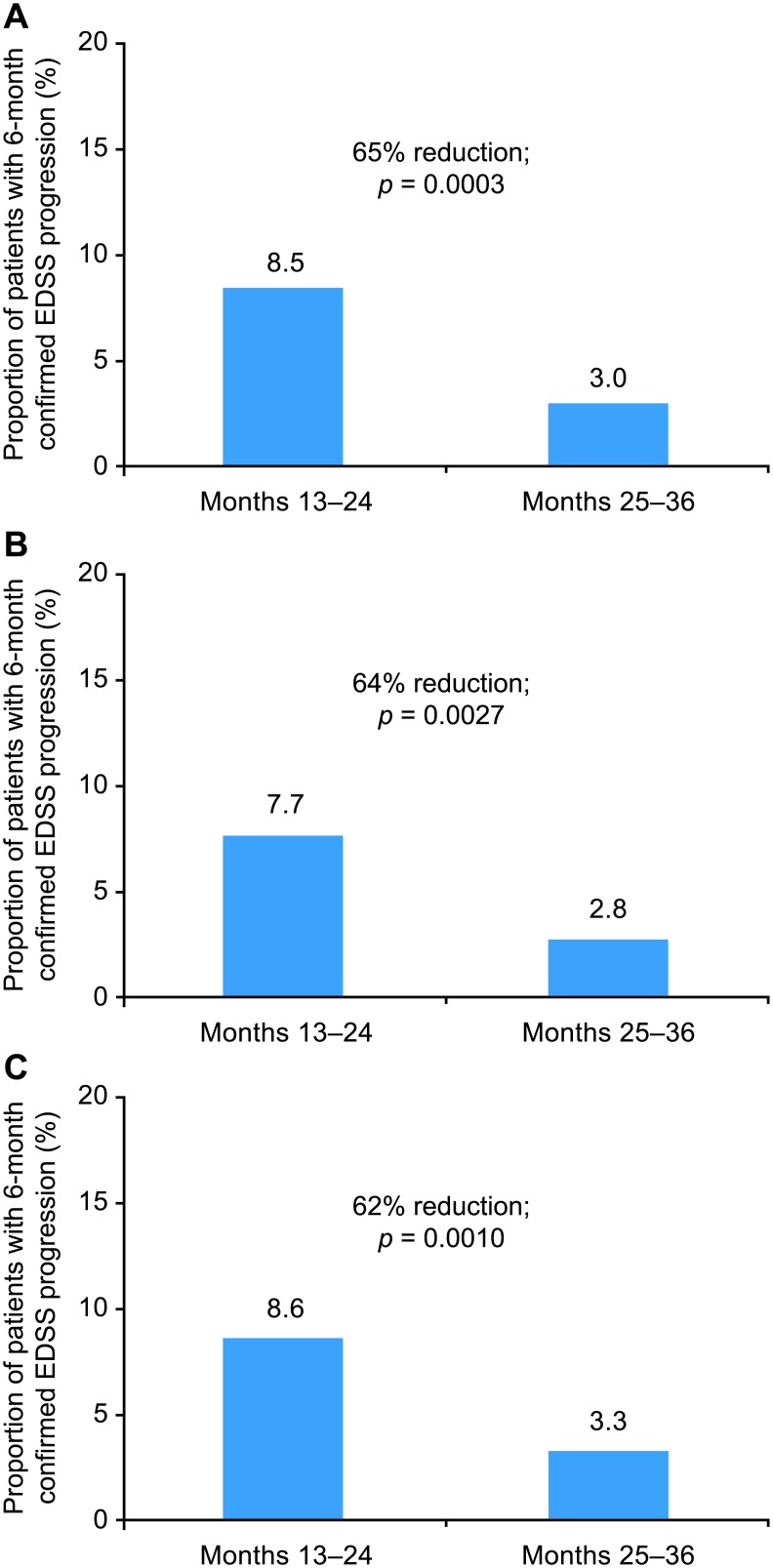
Sensitivity analyses of 6-month confirmed EDSS progression in months 13–24 compared with months 25–36: (A) with newly reset EDSS baseline (month 6–12) as a reference (n = 473), (B) excluding patients with relapse (n = 362), and (C) excluding patients with confirmed EDSS improvement (n = 337).

Additional sensitivity analyses (described in detail below), which were performed to determine whether the observed decrease in the rate of EDSS progression events occurring between months 13–24 and 25–36 of natalizumab treatment was dependent on the EDSS assessment used as baseline, on relapse activity, or on inclusion of patients with EDSS improvement, produced results consistent with the primary analysis. When an EDSS assessment between months 6 and 12 of natalizumab treatment was used as baseline, the rate of 6-month confirmed EDSS progression was reduced by 65% (*p* = 0.0003) between months 13–24 and 25–36 of treatment (Fig 6A). When patients with relapses were excluded from the analysis, the rate of 6-month confirmed EDSS progression was reduced by 64% (*p* = 0.0027) between months 13–24 and 25–36 of treatment (Fig 6B). When patients with 6-month confirmed EDSS improvement were excluded from the analysis, the rate of 6-month confirmed EDSS progression was reduced by 62% (*p* = 0.0010) between months 13–24 and 25–36 of treatment (Fig 6C).

In the 4-year completers, annualized relapse rate was low and remained low in each epoch (months 1–24, 0.19; months 25–48, 0.18; months 13–24, 0.18; months 25–36, 0.19).

Subgroup analysis in patients with baseline EDSS scores <3.0 (n = 184) showed that between months 13–24 and 25–36 of natalizumab treatment, rates of 6-month and 12-month confirmed EDSS progression were reduced by 73% (*p* = 0.0025) and 70% (*p* = 0.0060), respectively. In patients with baseline EDSS scores ≥3.0 (n = 312), confirmed EDSS progression rates were numerically lower in months 25–36, but there was no statistically significant difference between months 13–24 and 25–36 in 6-month confirmed EDSS progression (months 13–24, 4.8%; months 25–36, 2.9%; *p* = 0.2008) or 12-month confirmed EDSS progression (months 13–24, 4.2%; months 25–36, 2.6%; *p* = 0.2513).

## Discussion

Prior data from AFFIRM suggested that the effect of natalizumab treatment on achieving no evidence of clinical or radiological multiple sclerosis disease activity increased in the second year of treatment [2]. However, patients enrolled in the TOP study had more baseline disease activity, as measured by EDSS score and annualized relapse rate, than patients in either of the phase 3 trials of natalizumab (AFFIRM and SENTINEL) [1,7]. This post hoc analysis of TOP demonstrates in a clinical practice setting that the rate of confirmed disability progression in relatively severely affected RRMS patients was very low after initiation of natalizumab and further decreased beyond 2 years of natalizumab treatment, while relapse rates were consistently low across all treatment epochs. More specifically, rates of 6-month and 12-month confirmed EDSS progression were reduced by 42% and 52%, respectively, in months 25–48 compared with months 1–24; rates of 6-month and 12-month confirmed EDSS progression were reduced by 60% and 58%, respectively, in the months 25–36 treatment exposure window compared with the months 13–24 treatment window. Furthermore, patients with confirmed EDSS progression in the first 2 years of natalizumab treatment were very unlikely to experience another confirmed EDSS progression event in the subsequent 2 years.

One consequence of restricting our analyses to the 4-year completer population is that the conclusions may not be universally applicable. In particular, it is not known whether these findings would apply to patients who discontinue natalizumab treatment sooner because of efficacy concerns. However, there is some basis for thinking that the conclusions from this study may apply to the broader patient population, since (1) baseline characteristics were not different between the 4-year completers and the overall TOP study populations, (2) the proportion of patients with 6-month confirmed EDSS progression at 2 years did not differ significantly between the 4-year completer population and those who were expected to complete 4 years but discontinued natalizumab treatment after 2 years, and (3) mean EDSS scores during each of the 4 years of TOP were similar between the 4-year completer population and the overall TOP population.

Use of the 4-year completer population in TOP introduces 2 potential lines of bias. First, patients who remain on treatment for 4 years are likely experiencing benefits of treatment, which may make them content to remain on that therapy. In addition, although the rates of confirmed EDSS progression differed significantly between months 1–24 and months 25–48, caution is required when interpreting the results of the comparison between months 1–24 and months 25–48. Not all of the 4-year completer population had EDSS measurements beyond 4 years to allow for confirmation of EDSS progression events occurring in year 4. This potential confounding factor could impact both the 6-month and 12-month confirmed EDSS progression events, though in theory it would have a greater effect with a longer confirmation time. Our analysis comparing the month 13–24 and month 25–36 natalizumab treatment windows eliminated this potential confounding factor and also addressed the potential effect of variable loss of observation and treatment time in year 1 (caused by allowing up to 3 months of treatment prior to study enrollment). The rates of confirmed EDSS progression in months 13–24 and 25–36 remained significantly different when the period for progression confirmation was kept consistent across the epochs to avoid any potential bias introduced by differing lengths of observation following a progression event.

Natalizumab has previously been shown to lower relapse rates [1,8–11], decrease residual disability induced per relapse occurrence [12], and increase the proportion of patients with confirmed EDSS improvement [13]. Sensitivity analyses performed in this study indicate that the observed decrease in the rate of 6-month and 12-month confirmed EDSS progression events between months 13–24 and 25–36 of natalizumab treatment was independent of both relapse activity and EDSS improvement. Differences in the rates of progression events persisted when patients with relapses or EDSS improvement were removed from the analyses.

By including only patients who completed 4 years of natalizumab treatment in these analyses, we avoided a known bias of observational studies, which can overestimate treatment efficacy over time due to the selective discontinuation of partial responders or nonresponders [4–6]. Indeed, we showed that TOP patients who discontinued natalizumab due to reported lack of efficacy had a higher probability of EDSS progression in the first 2 years than the 4-year completer population; thus, their inclusion would have likely led to an overestimation of treatment efficacy increases with longer treatment time. However, it is important to note that among patients who were expected to complete 4 years of treatment but discontinued after 2 years of treatment (n = 514), only 17% of discontinuations were due to reported lack of efficacy, and EDSS progression rates during the first 2 years were consistently similar between the 4-year completer population and patients who discontinued for reasons other than reported lack of efficacy.

In this study, the increase in the effect of natalizumab on EDSS progression events over time appears to be strikingly independent of relapse activity, and thus it may not be fully explained by the same mechanisms that are responsible for the effects observed within the first 2 years of treatment, when natalizumab has been shown to reduce the formation of new active lesions in the central nervous system [1]. Disability progression in the absence of new lesion formation may be mediated by chronic active lesions, which are surrounded by a rim of macrophages that cause ongoing axonal injury [14,15]. As an inhibitor of alpha-4 integrin-expressing-cell migration through the blood-brain barrier [16], natalizumab may interfere with macrophage recruitment to these areas of chronic inflammation, reducing axonal injury and ultimately leading to reduced disability progression events. Thus, a plausible hypothesis to explain the current findings is that axonal injury in chronic active lesions is reduced with long-term natalizumab treatment, which may be associated with a decreased probability of disability progression events over treatment time, particularly in patients with EDSS scores <3.0 at baseline. Further analyses might seek to determine if this effect is associated with specific patient characteristics, such as age or disease duration, and if there is a corresponding increase of disability regression after EDSS progression events across these treatment epochs.

In summary, natalizumab has well-established efficacy in decreasing the risk of disability progression in the first 2 years of treatment [1,17,18]. In this study, we make the additional observation that, in patients who continue treatment beyond 2 years, the effect of natalizumab on disability progression appears to further increase with longer treatment times.

## Supporting Information

S1 Appendix TOP study co-investigators. (DOCX)

S1 Fig Patient disposition. (EPS)
