# Supplementary material for: Epoch Analysis of On-Treatment Disability Progression Events over Time in the Tysabri Observational Program (TOP)
Source: PLoS One. 2016 Jan 15;11(1):e0144834. doi: 10.1371/journal.pone.0144834 (PMC4714845; doi:10.1371/journal.pone.0144834)
Supplement: S1 Appendix — (DOCX) [file pone.0144834.s001.docx]

**TOP Coinvestigator List**

Argentina: Jorge Correale, Alejandro Caride, Norma H. Deri, Carlos Ballario. Australia: Simon Broadley, Chris Kneebone, Michael Barnett, John Pollard, Suzanne Hodgkinson, Allan Kermode, Richard Macdonell, John King, Helmut Butzkueven, Jeannette Lechner-Scott, Noel Saines, Mark Slee, Chris Plummer. Belgium: Barbara Willekens, Ludo Vanopdenbosch, Shibeshih Belachew, Rémy Phan-Ba, Valérie Delvaux, Veronique Bissay, Jan Debruyne, Danny Decoo, Roeland Crols, Anoek Symons, Guy Nagels, Vincent Van Pesch, Christian Sindic, Benedicte Dubois, Robert Medaer, Marie D'Hooghe, Daniel Guillaume, Eric De Smet, Pierrette Seeldrayers, Andreas Lysandropoulos, Mathieu Vokaer, Karine Geens, Christina Willems, Pierre Denayer, Michel Bureau, Cecile Retif, Michel Dupuis, Olivier Bouquiaux, Patrick Vanderdonckt, William van Landegem, Jo Caekebeke, Erwin Van Ingelghem, Katelijne Peeters, Pascale Gerard, Alain Maertens de Noordhout, Philippe Desfontaines, Etienne Urbain, Inge Declercq, Bart Van Wijmeersch, Erwin Vanroose, Alain Wibail, Emmanuel Barthomolé. Canada: Melanie Ursell, Margaret Elizabeth Sweet, David Howse, Draga Jichici, Melad Shawush, Mike Namaka, Anthony Traboulsee, Stan Hashimoto, Raymond Lo, Paul Marchetti, Yves Lapierre, Francois Jacques, Gregg MacLean, Virender Bhan, Pierre Duquette, Bradley Stewart, John Paulseth, Marcelo Kremenchutzky, Galina Vorobeychik, Paul O'Connor, François Grand'Maison. Czech Republic: Eva Havrdova, Eva Meluzinová, Martin Valis, Radomír Talab, Pavel Stourac, Olga Zapletalová, Michal Dufek, Vladimíra Sládková, Alena Novotna, Romana Vancurová, Libuse Lhotaková, Jiri Fiedler, Marta Vachova, David Dolezil, Ivana Stetkarova, Adela Rehankova, Petr Psenica, Veronika Ulehlova, Sona Feketova, Ondrej Skoda. Finland: Markus Färkkilä, Sarasoja Taneli, Keijo Koivisto, Juha Matti Seppä, Laura Airas, Irina Elovaara, Päivi Hartikainen, Tuula Pirttila. France: Pierre Louchart, Olivier Ille, Jean philippe Thenint, Etienne Godet, Marcel Maillet Vioud, Renato Colamarino, Michel Gugenheim, Jerome Grimaud, Audrey Kopf, Christophe Billy, Bernard Huttin, Jean paul Borsotti, Philippe Devos, Jean bertin N Kendjuo, Albert Verier , Stephane Chapuis, Nathalie Daluzeau, Gilles Angibaud, Marie-Sylvie Artaud Uriot, François Ziegler, François Sellal, Antoine Moulignier, Isabelle Lavenu, Samir Ismail, Richard Devy, Manuel Suceveanu, Marc Wagner, Sebastien Marcel, Faycal Derouiche, Sohrab Mostoufizadehghalamfarsa, Sophie Delalande, Irene Ruggieri, Catherine Bossu Van Nieuwenhuyse, Chantal Nifle, Basile Ondze, Carmen Gurau Vasilescu, Cyrille Vongsouthi, Marc Coustans, Olivier Anne, Josephine Amevigbe, Jerome Servan, Marc Merienne, Philippe Eck, Stephane Berroir, Philippe Busson, Bruno Barroso, Jean-Marc Larrieu, Catherine Louvet Giendaj, Imad Malkoun, Patrick Hautecoeur, Arnaud Kwiatkowski, Andre Pouliquen, Guillaume Garrigues, Olivier Delerue, Pierric Giraud, Julien Gere, Jean Vaunaize, Olivier Dereeper, Nicolas Seiller, Roger Alsassa, Mihaela Vlaicu, Veronique Neuville, Jean Marc Faucheux, Patricia Bernady, Guy Fanjaud, François Viallet. Denmark: Michael Schroeter, Sylke Schlemilch-Paschen, Thomas Lange, Kin-Arno Bohr, Klaus Jendroska, Elisabeth Rehkopf, Arnfin Bergmann, Christoph Kleinschnitz, Thomas Postert, Peter Scholz, Uwe Mauz, Hubert Stratmann, Veneta Siefjediers, Martin Prantl, Klaus Gehring, Ruth Zellner, Kathrin Junge, Anton Zellner, Valerina Bacay, Eugen Schlegel, Udo Polzer, Erik Strauss, Andreas Link, Christoph Stenzel, Matthias Freidel, Joachim Drews, Christian Neudert, Frank Schmitz, Joachim Jaeger, Said Masri, Wolfgang Heuberger, Beate Trausch, Oliver Ruhnke, Serena Scarel, Kathlen Bach, Michael Ernst, Harald Landefeld, Nils Richter, Stephan Schmidt, Michaela Krause, Alezander Dressel, Roland Ruth, Kerstin Anvari, Jens Gossling, Christoph Schenk, Oliver Tiedge, Lutz Bode, Hans-Thomas Eder, Oliver Pfeffer, Reinhard Krug, Christoph Lassek, Eberhard Fleischer, Sven Meuth, Luisa Hildegard Klotz, Ines Peglau, Borries Kukowski, Birgit Herting, Kersten Guthke, Jurgen Schierenbeck, Bernd Brockmeier, Holger Albrecht, Matthias Wuttke, Regine Augspach-Hofmann, Stefan Gunther, Martin Redbrake, Christian Franke, Klaus Buchner, Thomas Gratz, Rolf Horn, Frank Doemges, Martin Schreiber, Thomas Brosch, Markus Horn, Matthias Kittlitz, Gabriele Vulturius, Paul Hinse, Rolf Malessa, Stephan Wiehler, Zaza Katsarava, Oliver Kastrup, Ulrich Kausch, Martin Gullekes, Markus Fickinger, Wilhelm Wenzel, Ingolf C. Botefur, Gerd Reifschneider, Sebastian Rauer, Michael Lang, Lutz Harms, Ulrich Eckhardt, Simone Cursiefen, Ralf Linker, Klemens Angstwurm, Judith Haas, Ivo Schuetze, Eva Rohm, H. Stienker-Fisse, Michael Sailer, Johannes Bohringer, Mathias Maurer, Eberhard Bause, Ronald Wersching, Reinhardt Dachsel, Sylke Domke, Frank Hoffman, Bjorn Tackenberg, Kerstin Roch, Uwe Ziebold, Boris Kallmann, Bernhard Buehler, Judith Faiss, Juergen Faiss, Sebastian Schimrigk, Christian Menges, Karl Christian Knop, Wolfgang Koehler, Arno Siever, Johannes Bufler, Georg Gramsl, Benedicta Kuhnler, Matthias Maschke, Florian Stogbauer, Lisa Staude, Florian Bethke, Andreas Bitsch, Arndt D. Harmjanz, Jorg Windsheimer, Bernd C. Kieseier, Ralf Berkenfeld, Hayrettin Tumani, Michael Kirsch, Brigitte Wildemann, Regina Daniels, Klaus Gottwald, Wolfgang-Gerhard Elias, Olaf Hoffmann, Matthias Schwab, Christopher Pilz, Fabian Klostermann, Kerstin Hellwig, Achim Berthele, Antonios Bayas, Daniel Molitor, Christoph Grothe, Bert Wagner. Greece: Klimentini Karageorgiou, Dimosthenis Mitsikostas, Antonios Kodounis, Andreas Plaitakis, Alexandros Papadimitriou, Nikolaos Grigoriadis, Nikolaos Vlaikidis, Evaggelos Koutlas, Athanassios Kyritsis, Panagiotis Papathanassopoulos, Nikolaos Makris, Antonios Tavernarakis. Italy: Elio Scarpini, Enrico Montanari, Maria Giovanna Marrosu, Maria Trojano, Maria Pia Amato, Mariarosa Rottoli, Alessandra Lugaresi, Ciro Florio, Claudio Gasperini, Luigi Grimaldi, Enrico Millefiorini, Tatiana Koudriavtseva, Franco Perla, Renato Mantegazza, Antonio Bertolotto, Angelo Ghezzi. Mexico: Sandra Quinones Aguilar, Eli Skromne Eisenberg, Leondardo Llamas Lopez, Rocio Marquez Estudillo. Netherlands: H.M. Schrijver, M.C. Wittebol, J.C. Baart, A.E.L. van Golde, G.J.D. Hengstman, P.H.M. Pop, M. Bos (Geldrop), R. Medaer, Angelique Schyns-Soeterboek, A. van der Zwart, A.J.H. van Diepen, G.A.M. Verheul, W.I.M. Verhagen, M. Bos (Helmond), R.J.G.M. Witjes, L.G.F. Sinnige, E. Th. L. van Munster, E.A.C.M. Sanders, Ron van Dijl, R.M.M. Hupperts, S.T.F.M. Frequin, L.H. Visser, J.M.L. Henselmans, J.W.B. Moll. Norway: Rune Midgard, Kjell Morten Myhr, Astrid Edland, Wenche Telstad, Tone Hognestad, Christian Lund, Harald Hovdal, Kaur Kamaljit, Jan Schepel, Roelfien Ida Hogenesch, Stephan Schüler, Francis Odeh, Karl B. Alstadhaug, Olav Korsgaard, Elisabeth Farbu, Teis Barclay Ingvaldsen. Portugal: Diana Soares (SCO), José Rente, José Manuel Costa Guerra, Armando Morganho, António Leitão, João de Sá, Maria José Sá, Pinto Marques, Mário Veloso, Miguel Viana Baptista. Slovakia: Jarmila Szilasiová, Daniela Copikova-Cudrakova, Lubica Prochazkova, Eleonóra Klimová, Vladimir Donath, Miroslav Brozman. Spain (ES): Cristina Ramo, Domingo Pérez Ruiz, Carmen Calles Hernández, María Eugenia Marzo Sola, Roberto Suarez Moro, Jose Antonio Vidal, Ana Belén Caminero Rodríguez, Gisela Martin Ozaeta, Jordi Batlle Nadal, Amaya Alvarez de Arcaya Esquide, Javier Olascoaga Urtaza, Sergio Martínez-Yélamos, Txomin Arbizu, Lluis Ramio i Torrenta. Great Britain: Mike Boggild, Martin Wilson, Adnan Al-Araji, Richard Nicholas, Timothy Harrower, Ian Redmond, Tilo Wolf, Michael Osei-Bonsu, Gordon Mazibrada, David Rog, David Cottrell, Cris Constantinescu, Orla Gray, Mohamed Belhag, Abdullah Shehu, Waqar Rashid, Martin Duddy.
